# Prevalence of cramps in patients over the age of 60 in primary care : a cross sectional study

**DOI:** 10.1186/s12875-016-0509-9

**Published:** 2016-08-12

**Authors:** Hubert Maisonneuve, Juliette Chambe, Chloé Delacour, Joris Muller, Fabien Rougerie, Dagmar M. Haller, Michel Leveque

**Affiliations:** 1 Primary Care Unit, Faculty of Medicine, University of Geneva, rue Michel Servet 11, 1211 Geneva, Switzerland; 2 General Medicine Department, Faculty of Medicine, University of Strasbourg, 4 rue Kirschleger, 67000 Strasbourg, France; 3 Public Health Department, Faculty of Medicine, University of Strasbourg, 4 rue Kirschleger, 67000 Strasbourg, France

**Keywords:** Nocturnal leg cramps, Prevalence, Primary care, General practice

## Abstract

**Background:**

Cramps are involuntary painful muscle contractions that mainly affect older people. Cramps may cause severe pain and sleep disturbance. Little information exists on the prevalence and the main features of cramps in primary care settings. The objective of this study was to estimate the prevalence and the main features of cramps among primary care patients aged 60 years and older.

**Methods:**

We prospectively enrolled 516 patients aged 60 years and older in a cross-sectional study at 25 general practices in Alsace – France between October 2011 and March 2012. Questionnaires were used to obtain information about demographics, cramp presence and main features, medical history, and treatment.

**Results:**

The adjusted prevalence was 46 % (95 % CI: 38–53 %). Thirty-one per cent of the study sample reported being woken up by cramps, 15 % had cramps more than 3 times a month. Logistic regression revealed a slightly higher prevalence in the age group 65–69 years compared to 60–64 years. No significant association was observed between other age groups and prevalence, or between gender and prevalence. The main localization of cramps was in the calves (80 %). The duration since onset was 5 years or more for 58 %.

**Conclusions:**

Cramps are common in primary care, and although only a minority of patients report suffering from cramps more than once a week, many patients report cramp-related sleep disturbance. Further studies are needed to assess risk factors and therapeutic options for patients suffering from cramps in primary care.

**Electronic supplementary material:**

The online version of this article (doi:10.1186/s12875-016-0509-9) contains supplementary material, which is available to authorized users.

## Background

A cramp is defined as a sudden, involuntary painful contraction of a muscle causing a palpable knot in the muscle [1]. Cramps last a few seconds to a few minutes and ease spontaneously [1]. Stretching or contraction of the antagonist muscle usually speeds relief [2]. Parisi’s classification describes 3 entities: (1) idiopathic cramps with nocturnal leg cramps, (2) paraphysiological cramps with pregnancy related cramps and exercise induced cramps and (3) symptomatic cramps related to etiological factors such as medication or medical conditions [3, 4]. Most cramps occur during periods of rest, mainly during the night [5].

The gastrocnemius, soleus and plantar muscles are the most susceptible to cramps. Cramps mainly affect older people [6] with a mean age of onset of 60 years [7].

Cramps are an overlooked but clinically meaningful event. Reports from a United States veteran outpatient clinic and a United Kingdom primary care setting indicate 37 to 56 % of patients are affected [6, 7].

In a study involving 233 patients suffering from cramps, selected from a general practice register, patients reported an important impact of night cramps on their quality of life. Their symptoms were evaluated as a “major nuisance” for 26 % of patients, and as “very distressing” for 24 % [7].

Most studies to date were conducted more than 20 years ago, in hospital and/or specialized settings and were limited by small sample sizes. One exception is Naylor’s study, which was conducted in primary care, but the participants were selected from only one general practice register [7]. Also, all of these studies used self-administered questionnaires; a method likely to introduce selection bias and uncertainty regarding the term *cramp* which is often improperly used to indicate various muscular manifestations [3]. To our knowledge, no previous study has examined both prevalence and the main features of cramps suffered by patients attending various primary care practices. Given the high impact of cramps on patients’ quality of life, there is a need to provide data on their epidemiology in the context of primary care. Thus, the research question for this study was:

What are the prevalence and the main features of cramps in people aged 60 and older in a primary care setting?

## Methods

### Design

This observational cross-sectional study was conducted in general practices in France. We used the STROBE statement to guide the reporting of our study.

### Participants, centres

The study was conducted within the Strasbourg General Medicine Department practice based research network (GMD PBRN) between October 2011 and March 2012. This network included 25 general practices spread all over Alsace region. They enrolled patient aged ≥60 years attending the practice of their general practitioner (GP). Patients were sampled by the GPs using a systematic step of 1 in 3 attending patients aged ≥60 years.

Inclusion criteria were: patient aged ≥60 years, speaking French, capable of full independent living, physically able to go to the practice, having signed an informed consent. Exclusion criteria were: patient not speaking French and unable to give informed consent. As the pathophysiological mechanism of exercise cramps is different, we did not consider people with cramps occurring only after or during exercise as belonging to the cramp group.

To evaluate whether our sample was representative of the population of patients ≥60 years attending in General Practice in Alsace, we compared it to a population of patients aged between 60 and 90 years, consulting between October 2011 and March 2012. These data were extracted from the regional health insurance database.

### Outcome measures

We developed and piloted a two-sections questionnaire, using information from several reviews on the topic to create the questions [4–11]. The questions focused on demographics, the presence and main features of cramps, medical history, clinical examination, and treatment.

The questionnaire was explicit in defining cramps as a painful involuntary muscle contraction when resting, lasting from a few seconds to a few minutes. We followed Parisi’s choice of Layzer’s clinical definition: “spasmodic, painful, involuntary, contraction of the skeletal muscle”, or in non-medical language: “sudden, involuntary and painful muscular contraction” [3]. This definition seems to be accepted by most of the authors [1–8, 10, 12]. If in doubt with regard to whether the patient truly had cramps, GPs were advised to explore another typical feature of cramps i.e. whether stretching and contraction of the antagonist muscle speeds relief [2, 8].

The GPs were all trained for the inclusion process and questionnaire administration. The questionnaire was administered by the GPs during the consultation time.

In the first section, the GP asked patients about the presence, laterality, localization, frequency and onset of the cramps. Questions were also asked about whether the cramps were occurring at night, and were waking the patients up.

The second section concerned only the cramps sufferers.

The physician checked the patient’s medical record to obtain information about his/her medical history, cramp-specific medication, general medication and performed a medical examination. Specific conditions known to be associated with cramps [3] were sought: high blood pressure, peripheral arteriopathy, chronic venous insufficiency, diabetes, severe to terminal renal insufficiency, peripheral neuropathy, restless leg syndrome, spinal stenosis and physical activities. We chose to collect information concerning these conditions in order to compare their prevalence within our sample of cramp sufferers with the prevalence of these conditions in a population of patient without cramps. The control population was extracted from the database of another study conducted in 2013 in the GMD PBRN aiming to evaluate the association between sedentary lifestyle and nocturnal leg cramps [13, 14].

The English version of the questionnaire is available in Additional file 1.

### Data analysis

We computed overall prevalence of cramps, cramps occurring more than 3 times a month, and cramps that led to awakenings. Results are reported with 95 % confidence intervals adjusted for clustering within practices.

We computed proportions of medical conditions and general medication known to be associated with cramps (anti-hypertensive treatment, lipid-lowering treatment, beta mimetic) [10] and compared them with data collected from patients not experiencing cramps within a similar population, as part of a study conducted in 2013 in the GMD PBRN. We performed chi-square tests to compare the gender and age groups between our sample and the health insurance population and to compare the proportions of medical conditions and general medications between our sample of cramp sufferers and our sample of patients not experiencing cramps.

A logistic regression analysis adjusted for clustering within practices was performed to examine the association of cramps with age and sex.

Stata 12 was used for all statistical analyses.

## Results

### Flow of participants

Of 52 eligible members of the GMD PBRN, 26 (50 %) agreed to participate, 25 (48 %) took part in the entire study. The survey was proposed to 549 patients. Among them, 516 agreed to participate (participation rate 94 %).

Of the participants, 57 % were women compared to 56 % in the health insurance population (no significant difference for gender, *p* = 0,62). Mean age was 71 years (SD 7,46) for our sample and 71 (SD 6,38) for the regional health insurance population. Table 1 shows the comparison of the age groups between our sample and the health insurance population. Overall, patients between 65 and 80 years old were slightly overrepresented among patients in our study.

**Table 1:** Comparison between study population and reference population for age group

Age	Study population %, (*n* = 516)	Reference population %, (*n* = 393796)	p
60–64	25, (129)	29, (114687)	0.04
65–69	20, (101)	19, (75646)	<0.01
70–74	20, (102)	18, (69321)	0.19
75–79	21, (110)	16, (61587)	<0.01
>80	14, (74)	18, (72555)	0.02

### Prevalence

Table 2 shows the total prevalence and distribution according to gender and age. The prevalence of cramps was 46 % in our setting. Logistic regression revealed a discretely higher prevalence in the age group 65–69 compared to 60–64. No significant association was observed between other age groups and prevalence, nor between gender and prevalence. Additional file 2 details the prevalence of cramps among the study population.

**Table 2:** Total cramp prevalence and distribution according to gender and age

Characteristics	N	Cramps % (95 CI)
Total	516	46 (38 to 53)
Gender		
Female	295	46 (40 to 52)
Male	221	45 (39 to 52)
Age		
60–64	129	40 (31 to 48)
65–69	101	52 (43 to 62)
70–74	102	52 (43 to 63)
75–79	110	45 (35 to 54)
> 80	74	41 (29 to 52)

### Main features

#### Awakening and frequency

**Fig. 1:**
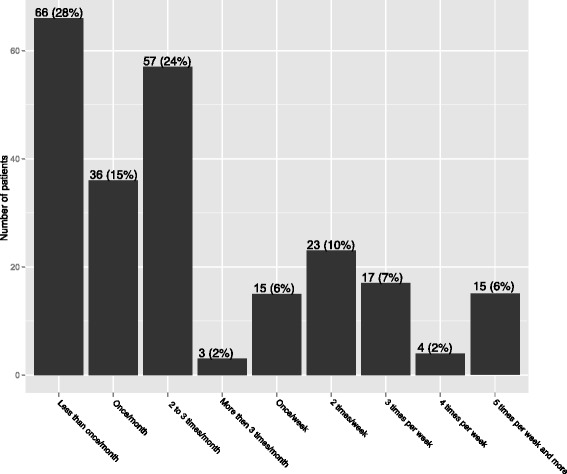
Distribution of cramp frequency: number and percentage of patients allocated by cramp frequency

Table 3 shows the adjusted prevalence for awakening cramps and frequent cramps. Thirty one per cent (*n* = 161) of the entire study sample reported being woken up by cramps. Figure 1 shows the distribution of cramp frequency. Among the patients suffering from cramps, 67 % had cramps less than 3 times a month, and 23 % between once and 3 times a week.

**Table 3:** Adjusted prevalence for cramps, cramps related with sleep disturbance and >3/month cramps

Cramp characteristics	Adjusted^a^ prevalence in % (95 CI) *N* = 516
Cramps	46 (38 to 53)
Cramps related to sleep disturbance	31 (26 to 36)
Cramps > 3/month	15 (10 to 19)

^a^Adjustment for clustering within general practices

Additional file 3 details the occurrence of cramps for each cramps sufferer among the study population.

### Medical history, medication, localization and date of onset

Table 4 shows the comparison of medical conditions and treatments between our cramp sufferers’ sample and the sample of patients not suffering from cramps from our reference population. Additional files 4 and 5 show the patients’ detailed medical history and treatments. There was a significant difference between the two populations for venous insufficiency, peripheral arteriopathy and restless leg syndrome. There was no significant difference between the two populations for antihypertensive, lipid-lowering and beta mimetic agents. The most common localization of cramps was in the calves and most patients (58 %, 95CI: 52 to 65 %) reported that they had been suffering from cramps for more than five years (see Additional file 6 for detailed report of onset).

**Table 4:** Comparison of main features related to cramps in people aged 60 and older

Characteristics	Occurrence in patients with cramps *n* = 236 (%)	Occurrence in patients without cramps (comparative sample) *n* = 185 (%)	*P*
Medical condition			
Hypertension	153 (65)	128 (69)	0.35
Diabetes	46 (19)	31 (17)	0.47
Venous insufficiency	71 (30)	6 (3)	<0.001
Peripheral arteriopathy	37 (16)	4 (2)	<0.001
Hypothyroidism	24 (10)	12 (6)	0.18
Restless leg Syndrome	16 (7)	0 (0)	<0.001
Peripheral neuropathy	10 (4)	4 (2)	0.24
Severe to terminal renal insufficiency	9 (4)	8 (4)	0.79
Current drug treatment			
Anti hypertensive	151 (64)	123 (66)	0.59
*Thiazides*	67 (28)	53 (29)	0.95
*ACE/ARBs* ^a^	114 (48)	87 (47)	0.79
*Beta-blockers*	72 (30)	53 (29)	0.75
*Calcium Channel Blockers*	59 (25)	40 (22)	0.41
*Loop diuretics*	22 (9)	9 (5)	0.08
*Potassium Spare Diuretics*	21 (9)	12 (6)	0.36
*Central-acting agents*	8 (4)	5 (3)	0.65
Lipid-lowering	111 (47)	74 (40)	0.14
*statin*	104 (44)	72 (38)	0.28
*fibrates*	7 (3)	2 (1)	0,18
Beta-mimetics	21 (9)	10 (5)	0.17
Quinine	8 (3)	NC	
Localization			
Calves	190 (80)	NC	NC
Thigh	72 (30)	NC	NC
Feet	36 (15)	NC	NC
Others	4 (2)	NC	NC

^a^Angiotensin Converting Enzyme Inhibitors/Angiotensin receptor blockers

## Discussion

### Main findings

Nearly half the patients indicated suffering from cramps. Nearly one third of the patients declared being woken up by cramps. Although the majority of those who had cramps had had them for more than five years, two thirds of them reported infrequent episodes, less than three times a month. Gender was not associated with cramp prevalence.

### Prevalence and main features

In contrast to earlier studies, we conducted this survey in 25 distinct practices. Yet our results are consistent with previous estimations of prevalence from a single clinic or practice [6, 7]. This study confirms that cramps are a frequent problem in primary care. In contrast to a previous study by Naylor & Taylor in the United Kingdom [6], we found a weak association between age and cramps. A possible explanation for this may be the small subgroups in Naylor & Taylor’s study (between 13 and 33 patients) and the lack of statistical analysis. These results are reinforced by the date of onset of more than 5 years in most cases in both studies. Oboler et al. reported that a quarter of their veteran population described having cramps more than five times a week, a much higher proportion than in our study, in which only 6 % of patients indicated having cramps as frequently [6]. This rather contradictory result may be explained by the differences in setting, population and perhaps, seasonality between the two studies [15]. However, the difference cannot be attributed to the use of Quinine Sulfate (10 % in Oboler’s study and <5 % in ours). The low number of patients treated with quinine in our population may be explained by a national recommendation of discontinuation and a cessation of the reimbursement of Quinine in 2011. Our findings confirm that gender is not associated with cramp prevalence; as previously shown for children, young adults and the aged [7, 16]. We found a significant difference between our cramp sufferer sample and our reference population for the presence of venous insufficiency, peripheral arteriopathy and restless leg syndrome. The association between nocturnal leg cramps and venous insufficiency or arteriopathy had already been shown in various studies [6, 7, 17–19]. The association between nocturnal leg cramps and restless leg syndrome could be spurious [20], as patients may find it difficult to differentiate restless leg syndrome from cramps, leading to measurement bias [6].

Associated medical conditions in our sample of patients with cramps differed slightly from those of patients in Oboler et al’s study. There is more hypertension but less coronary artery disease, and stroke. This result may be explained by the difference between our primary care sample and male veterans in the Oboler et al. study.

### Complexity of the definition

As patients may use the word “cramp” to describe a large variety of symptoms, we had to focus on the definition of cramps we wanted to refer to. A relevant clinical definition was needed to bring investigators and recruited patients to a discussion on the same precise problem. The complexity of this definition is a major issue for researchers as well as investigators. Explanations to patients are likely to be investigator-dependent and may easily become time consuming. We tried to reduce this bias by reviewing the literature to design our questionnaire and by training the participant GPs, thus favoring a more homogeneous data collection compared to the use of self-administered questionnaires such as in Naylor’s or Oboler’s studies. Despite these limitations, our study supports the need for a precise definition of nocturnal leg cramps, which deserve to be explored more specifically in further studies [20]. The development and validation of a standardized questionnaire to explore nocturnal leg cramps could be a useful research tool in this field.

### Sleep, quality of life

Naylor’s study showed a high impact of cramps on quality of life [7]. In an Australian study, individuals who experienced nocturnal muscle cramps had significant lower health-related quality of life for the SF36 domains role physical (−19 points), bodily pain (−11 points) and general health (−10 points). This impact was largely explained by the negative impact of cramps on quality of sleep [21]. This is consistent with our findings that cramps awaken 31 % of our entire general practice sample.

Patients suffering from cramps experience more or less long periods of time without symptoms. This highlights potential difficulties in evaluating the impact of cramps on quality of life between symptomatic and non-symptomatic periods. The consequence on the treatment of this specific presentation of symptoms is also important. Patients are likely to favour a treatment that provides rapid and long-lasting relief. Considering the pathophysiology of cramps [8] and the association with specific medical conditions, this may not be a realistic goal. To date, none of the treatments used to treat cramps have been shown to be both effective and safe [5, 22]. More research is needed in this field.

### Local generalizability of our findings

Participation rate was high, (94 %) suggesting patients are interested in this theme and reinforcing the value of studying cramps in primary care. Overall, age and gender were similar in our sample compared to the general practice population in our region, although patients between 65 and 80 years were slightly overrepresented. This minimal difference may in part be due to the fact that we did not include bedridden patients or patients with limited mobility. Despite these slight differences, our findings seem representative of a population of patients consulting GPs in our region.

### Limitations

Our study was conducted in only one French region, in 25 practices, members of a regional research network. Although our results are consistent with the literature, replication of our study in other settings would be useful to assess the extent to which they are generalizable. Our age groups differ slightly from the reference population, but seem representative of a population of patients attending GPs. Although we constructed our questionnaire from a review of literature, we did not validate it, and therefore cannot exclude some level of measurement bias. The medical conditions and medications were extracted from the GPs’ medical records. The extent to which these were complete is unknown. As we had not collected clinical data for patients not experiencing cramps in the study, we tried to compensate for this weakness by comparing our sample to another sample extracted from the same setting.

### Implication for practice and research

The findings of this study have a number of important implications for practice. The high frequency of cramps and their association with sleep disturbance highlights the importance for physicians to ask their patients about cramps before prescribing symptomatic treatments for insomnia. Physicians should also be aware of the association between cramps and peripheral arteriopathy or chronic venous insufficiency. As cramps are easily confused with restless leg syndrome, a precise exploration of symptoms is essential.

This study emphasizes the need to develop and validate a tool for assessing nocturnal leg cramps, a better exploration of the consequences of cramp frequency variations on quality of life, a better identification of the risk factors, and an exploration of patients’ treatment expectations.

## Conclusion

Cramps are common in primary care, and although only a minority of patients report suffering from cramps more than once a week, many patients report cramp-related sleep disturbance. Future research should provide guidance about the risk factors associated with cramps, and explore patients’ expectations about treatment. More research is also needed to identify effective treatments for this common, yet much neglected disorder.

## Additional files

Additional file 1: English version of the CIPA Prevalence questionnaire. English translation of the complete questionnaire used during the study to identify cramps sufferers among the study population. (DOCX 76 kb) Additional file 2: AF Table 1: detailed prevalence of cramps in the study population. Detailed presence of cramps, cramps related with sleep disturbance, cramps more frequent than 3 times a month, gender and age-group among the study population. (CSV 8 kb) Additional file 3: AF Table 2: detailed occurrence of cramps. Detailed occurrence for each cramps sufferer of the study population. (CSV 5 kb) Additional file 4: AF Table 3: detailed medical history. Detailed medical history of each patient focussed on medical conditions at risk of cramps. (CSV 9 kb) Additional file 5: AF Table 4: detailed treatment. Detailed treatment of each patient, reporting also phytotherapy and homeopathy, without targeting medication at risk of cramps. (CSV 27 kb) Additional file 6: AF Table 5: duration of cramps detailed for each cramps sufferer of the study population. Date of onset of cramps specified for each patient reporting cramps. (CSV 5 kb)

## Abbreviations

GP: General Practitioner
GMD PBRN: Strasbourg General Medicine Department Practice Based Research Network
