# Supplementary material for: Prevalence of cramps in patients over the age of 60 in primary care : a cross sectional study
Source: BMC Fam Pract. 2016 Aug 12;17:111. doi: 10.1186/s12875-016-0509-9 (PMC4983045; doi:10.1186/s12875-016-0509-9)
Supplement: Additional file 1: — English version of the CIPA Prevalence questionnaire. English translation of the complete questionnaire used during the study to identify cramps sufferers among the study population. (DOCX 76 kb) [file 12875_2016_509_MOESM1_ESM.docx]

**CIPA PREVALENCE QUESTIONNAIRE**

**DEMOGRAPHICS**

**Age**

**Gender**

**PRESENCE AND MAIN FEATURES OF CRAMPS**

**Do you suffer from cramps?**

Definition: Spasmodic, painful involuntary muscle contraction when resting, lasting from a few seconds to a few minutes. If in doubt, ask the patient whether stretching and/or contraction of the antagonist muscle speeds relief

Yes

No

**Have you been suffering from cramps for more than one month?**

Yes

No

**Cramps are there from both sides or one side**

Both sides

One side

**Where are located you cramps?**

Calves

Feet

Thigh

Other

**Do they appear during the night?**

Yes

No

**Do cramps awake you or are related with sleep disturbance?**

Yes

No

**In an average month, how many times do you suffer from cramps?**

*If more than 3 times/month:*

**In an average week, how many times do you suffer from cramps?**

*If more than 7 times/week*

**In an average night with cramps, how many times do you suffer from cramps?**

**Do you suffer from cramps more than 10 times a week?**

**How many years have you been suffering from cramps?**

*If less than one year:*

**How many months have you been suffering from cramps?**

**MEDICAL HISTORY**

**Medical conditions stated in the medical record**

**Cardiovascular:**

Hypertension

Arteriopathy

Venous insufficiency

**Endocrinology and metabolism:**

Diabetes

Hypothyroidism

Hyperthyroidism

Addison’s disease

Conn’s syndrome

**Nephrology**

Severe to terminal renal insufficiency

Dialysis

**Neurology and psychiatry**

Peripheral neuropathy

Multiple sclerosis

Amyotrophic lateral sclerosis

Parkinson’s disease

Restless legs syndrome

Alcohol addiction

**Oncology**

Active cancer

**Others**

**CLINICAL EXAMINATION**

**Blood pressure**

**Pulse**

**Symptoms of Left heart failure**

Yes

No

**Symptoms of right heart failure**

Yes

No

**Peripheral pulse**

**Anterior Tibial**

**Right** Yes No

**Left** Yes No

**Posterior Tibial**

**Right** Yes No

**Left** Yes No

**Symptoms of venous insufficiency:**

**None** Yes No

**Varicose veins** Yes No

**Ochre dermatitis** Yes No

**Lower limbs oedema** Yes No

**Lower limbs weakness**

Yes

No

**Amyotrophy**

Yes

No

**Fasciculation**

Yes

No

**Patellar reflex**

**Right** Yes No

**Left** Yes No

**Achilles reflex**

**Right** Yes No

**Left** Yes No

**Hypoesthesia to touch**

Yes

No

**Hypoesthesia to prick**

Yes

No

**In the painful area, can the pain be caused or increased by brushing?**

Yes

No

**TREATMENT**

**Do you take any treatment to relieve your cramps?**

Yes

No

*If yes:*

**Drug treatment:**

INN:

Proprietary name:

DDD:

Treatment initiation:

**Non-drug treatment:**

**Current drug treatment:**

**INN:**

Proprietary name:

DDD:

Treatment initiation:

DN4 Questionnaire:

**Does the pain have one or more of the following characteristics?**

Burning

Painful cold

Electric Shocks

**Is the pain associated with one or more of the following symptoms in the same area?**

Tingling

Pins and Needles

Numbness

Itching

None of these symptoms

**Does the pain oblige you to stop when you walk?**

Yes

No

*If yes*

**What is the minimum distance before you stop (in meters)?**

**If you stop, wait, and leave again, do you feel pain again after walking the same distance?**

**Do you practice a regular physical activity?**

Yes

No

**Can you precise?**
